# Supplementary material for: Contributions of Emotional Overload, Emotion Dysregulation, and Impulsivity to Eating Patterns in Obese Patients with Binge Eating Disorder and Seeking Bariatric Surgery
Source: Nutrients. 2020 Oct 12;12(10):3099. doi: 10.3390/nu12103099 (PMC7650699; doi:10.3390/nu12103099)
Supplement: Supplementary file 1 [file nutrients-12-03099-s001.pdf]

Supplementary material

Table 1: Results of regularized-method LASSO with inference

**Variables**	**Coefficient selected in the model**
Age	
Female/Male	
Emotional eating	**0.0001465112**
Restraint eating	
External eating	
BITE Symptom subscale	**0.2349467593**
BITE Severity subscale	
Beck Depression Inventory	
State anxiety inventory	
Trait anxiety inventory	
DERS Non-acceptance	
DERS Goals	
DERS Impulse	
DERS Awareness	
DERS Strategies	**0.0310587560**
DERS Clarity	

Table 2: Results of regularized-method LASSO with inference.

Variables	coefficients	Z-score	*p*-value	Low Conf Pt	Up Conf Pt	LowTail Area	UpTail Area
**Age**	0.063	1.358	***0.164***	-0.052	0.194	0.049	0.050
**Female/Male**	-0.992	-1.132	***0.204***	-5.231	1.529	0.049	0.049
**Emotional eating**	0.077	1.578	***0.060***	-0.009	0.545	0.049	0.050
**Restraint eating**	-0.032	-0.536	***0.316***	-0.629	0.284	0.050	0.050
**External eating**	0.006	0.094	***0.910***	-1.934	0.056	0.050	0.050
**BITE Symptom subscale**	0.343	2.661	***0.029***	0.057	0.906	0.049	0.049
**BITE Severity subscale**	0.107	0.715	***0.466***	-0.545	0.419	0.050	0.050
**Beck Depression Inventory**	0.065	0.722	***0.618***	-0.604	0.225	0.050	0.050
**State anxiety inventory**	-0.022	-0.532	***0.564***	-0.123	0.217	0.050	0.050
**Trait anxiety inventory**	-0.022	-0.383	***0.668***	-0.159	0.454	0.049	0.000
**DERS Non-acceptance**	-0.119	-1.162	***0.290***	-0.286	0.219	0.050	0.049
**DERS Goals**	-0.057	-0.455	***0.653***	-0.233	0.792	0.050	0.050
**DERS Impulse**	0.038	0.278	***0.680***	-1.457	0.555	0.050	0.050
**DERS Awareness**	-0.022	-0.242	***0.780***	-0.188	1.070	0.050	0.050
**DERS Strategies**	0.231	1.571	***0.170***	-0.202	0.721	0.050	0.049
**DERS Clarity**	-0.094	-0.674	***0.281***	-1.332	0.515	0.050	0.050

Table 3: Bayesian approach: Population-Level Effects

	Estimate	Est.Error	l-95% CI	u-95% CI	Rhat	Bulk_ESS	Tail_ESS
**Intercept**	-10.01	4.94	-19.98	-0.82	1.00	3441	3134
**Age**	0.09	0.05	-0.02	0.19	1.00	2691	2826
**Female/Male**	-1.19	0.99	-3.14	0.76	1.00	3765	3065
**Emotional eating**	0.10	0.06	-0.01	0.21	1.00	2970	3051
**Restraint eating**	-0.04	0.07	-0.18	0.09	1.00	3113	2821
**External eating**	0.01	0.08	-0.14	0.16	1.00	3428	2967
**BITE Symptom subscale**	0.45	0.15	0.19	0.77	1.00	2503	2248
**BITE Severity subscale**	0.16	0.17	-0.17	0.51	1.00	3822	2909
**Beck Depression Inventory**	0.08	0.10	-0.11	0.29	1.00	2901	2816
**State anxiety inventory**	-0.03	0.05	-0.12	0.06	1.00	3478	2712
**Trait anxiety inventory**	-0.03	0.07	-0.16	0.10	1.00	2957	2507
**DERS Non-acceptance**	-0.17	0.12	-0.42	0.05	1.00	2248	2448
**DERS Goals**	-0.06	0.14	-0.34	0.21	1.00	3539	3162
**DERS Impulse**	0.04	0.15	-0.27	0.34	1.00	2936	2620
**DERS Awareness**	-0.03	0.10	-0.23	0.17	1.00	3299	2937
**DERS Strategies**	0.32	0.17	0.00	0.68	1.00	2331	2512
**DERS Clarity**	-0.13	0.16	-0.46	0.18	1.00	2966	3054

Note. Samples: 4 chains, each with iter = 2000; warmup = 1000; thin = 1; total post-warmup samples = 4000.
